# New Cav1.2 Channelopathy with High-Functioning Autism, Affective Disorder, Severe Dental Enamel Defects, a Short QT Interval, and a Novel *CACNA1C* Loss-of-Function Mutation

**DOI:** 10.3390/ijms21228611

**Published:** 2020-11-15

**Authors:** Dominique Endres, Niels Decher, Isabell Röhr, Kirsty Vowinkel, Katharina Domschke, Katalin Komlosi, Andreas Tzschach, Birgitta Gläser, Miriam A. Schiele, Kimon Runge, Patrick Süß, Florian Schuchardt, Kathrin Nickel, Birgit Stallmeyer, Susanne Rinné, Eric Schulze-Bahr, Ludger Tebartz van Elst

**Affiliations:** 1 Section for Experimental Neuropsychiatry, Department of Psychiatry and Psychotherapy, Medical Center-University of Freiburg, Faculty of Medicine, University of Freiburg, 79104 Freiburg, Germany; kimon.runge@uniklinik-freiburg.de (K.R.); kathrin.nickel@uniklinik-freiburg.de (K.N.); tebartzvanelst@uniklinik-freiburg.de (L.T.v.E.); 2 Department of Psychiatry and Psychotherapy, Medical Center-University of Freiburg, Faculty of Medicine, University of Freiburg, 79104 Freiburg, Germany; katharina.domschke@uniklinik-freiburg.de (K.D.); miriam.schiele@uniklinik-freiburg.de (M.A.S.); 3 Institute for Physiology and Pathophysiology, Vegetative Physiology and Marburg Center for Mind, Brain and Behavior-Philipps-University Marburg, 35037 Marburg, Germany; decher@staff.uni-marburg.de (N.D.); info@isabell-roehr.de (I.R.); kirsty.vowinkel@staff.uni-marburg.de (K.V.); rinne@staff.uni-marburg.de (S.R.); 4 Center for Basics in Neuromodulation, Faculty of Medicine, University of Freiburg, 79106 Freiburg, Germany; 5 Institute of Human Genetics, Medical Center-University of Freiburg, Faculty of Medicine, University of Freiburg, 79106 Freiburg, Germany; katalin.komlosi@uniklinik-freiburg.de (K.K.); andreas.tzschach@uniklinik-freiburg.de (A.T.); birgitta.glaeser@uniklinik-freiburg.de (B.G.); 6 Department of Molecular Neurology, University Hospital Erlangen, 91054 Erlangen, Germany; Patrick.Suess@uk-erlangen.de; 7 Department of Neurology, Medical Center-University of Freiburg, Faculty of Medicine, University of Freiburg, 79106 Freiburg, Germany; florian.schuchardt@uniklinik-freiburg.de; 8 Institute for Genetics of Heart Diseases, Department of Cardiovascular Medicine, University Hospital Münster, 48149 Münster, Germany; Birgit.Stallmeyer@ukmuenster.de (B.S.); eric.schulze-bahr@ukmuenster.de (E.S.-B.)

**Keywords:** CACNA1C, CaV1.2, autism, short QT syndrome, dental enamel defect

## Abstract

Complex neuropsychiatric-cardiac syndromes can be genetically determined. For the first time, the authors present a syndromal form of short QT syndrome in a 34-year-old German male patient with extracardiac features with predominant psychiatric manifestation, namely a severe form of secondary high-functioning autism spectrum disorder (ASD), along with affective and psychotic exacerbations, and severe dental enamel defects (with rapid wearing off his teeth) due to a heterozygous loss-of-function mutation in the *CACNA1C* gene (NM_000719.6: c.2399A > C; p.Lys800Thr). This mutation was found only once in control databases; the mutated lysine is located in the Cav1.2 calcium channel, is highly conserved during evolution, and is predicted to affect protein function by most pathogenicity prediction algorithms. L-type Cav1.2 calcium channels are widely expressed in the brain and heart. In the case presented, electrophysiological studies revealed a prominent reduction in the current amplitude without changes in the gating behavior of the Cav1.2 channel, most likely due to a trafficking defect. Due to the demonstrated loss of function, the p.Lys800Thr variant was finally classified as pathogenic (ACMG class 4 variant) and is likely to cause a newly described Cav1.2 channelopathy.

## 1. Introduction

Autism spectrum disorders (ASD) are frequent neurodevelopmental disorders characterized by social interaction difficulties, stereotypical procedures, routines and rituals, or special interests caused predominantly idiopathic or of unknown cause (“primary forms”); in a subset, a specific cause can be identified, and some of these secondary cases (“secondary forms”) are part of genetically determined syndromes, e.g., fragile X syndrome [1]. Within these, Timothy syndrome is an extremely rare one characterized by long QT syndrome (LQTS) in the surface electrocardiography (ECG), skeletal abnormalities (e.g., cutaneous syndactyly), and neuropsychiatric features, such as autism [2,3,4], and is caused by gain-of-function mutations in the *CACNA1C* gene. Of note, some *CACNA1C* mutations may have an isolated cardiac, non-syndromal phenotype (only with QTc prolongation [5]). In contrast, patients with a short QT syndrome (SQTS) have not been described with extra-cardiac features so far.

## 2. Case Presentation

The authors present for the first time a syndromal form of SQTS in a 34-year-old German male patient that is characterized by extracardiac features, namely a severe form of secondary ASD together with affective and psychotic exacerbations, wearing of his teeth, and a short QT interval in the surface ECG due to a heterozygous loss-of-function mutation in the *CACNA1C* gene. The functional consequences of the genetic mutation were further analyzed using electrophysiological investigations of the mutant Cav1.2 calcium channel expressed in Xenopus oocytes [2]. The patient has given his signed written informed consent for this case report, including the presented images, the genetic information, and all other data, to be published.

### 2.1. Clinical Case Description

Since the first decade, the patient has suffered from the whole spectrum of autistic symptoms. The detailed diagnosis was performed according to the scheme established by the Freiburg Center for the Diagnosis and Treatment of Autism (http://www.uniklinik-freiburg.de/psych/live/patientenversorgung/schwerpunkte/schwerpunkt-asperger.html, accessed on 14 November 2020 [6,7,8]). His medical history showed alterations in (1) gaze control and holistic visual recognition (he had to learn to look into the eyes of others, uses analytical facial expression recognition, recognizes other people by certain characteristics); (2) problems with social communication (he lacks the tools to build relationships, is burdened by social contacts, small talk and telephoning cause him problems, plans social situations in detail in advance); (3) reduced social integration (has only a few close friends, needs his time alone); (4) interactional imagination abnormalities and sense of justice (he hated “doing things as if he were playing games”, his sense of justice is 10/10, he is very honest); (5) linguistic pragmatics (as a child, he read dictionaries to understand proverbs, is often interpreting things literally); (6) routines and rituals (plans his everyday life in detail, is stressed by changes of plan, usually eats and drinks the same things); (7) motor clumsiness (he always had difficulties in sports, he had two “left hands” and two “left feet”); (8) sensory hypersensitivity (for loud noises and light, tormented in former times already by stroking by the mother); (9) strong perception of details (e.g., finds comma errors in texts, has difficulties with the “overall picture”); (10) special memory capacity (always knows exactly where what stood when he had read something); and (11) special interests for computer science [6]. The Autism Diagnostic Observation Schedule, Second Edition (ADOS-2; at the age of the patient of 34 years) confirmed clear autistic communication and interaction behavior confirming a syndrome diagnosis (14 points; cut off > 10 [9]). The testing of the recognition of emotions (“gnosis facialis”) revealed indications of relative deficits in recognizing fear (http://www.gnosisfacialis.de/infoERT.html, accessed on 14 November 2020. In the Movie for the Assessment of Social Cognition (MASC), the patient scored borderline [10]. Difficulties in communication in social intuition were partially compensated by the patient in an analytical–cognitive way. In summary, two expert raters confirmed the presence of a high-functional ASD syndrome. His intelligence level was above average (he reached high school graduation “Abitur” in Germany with average grade 1.3; range: 1–6, optimal: 1, worst: 6) and later studied computer science.

**Figure 1:**
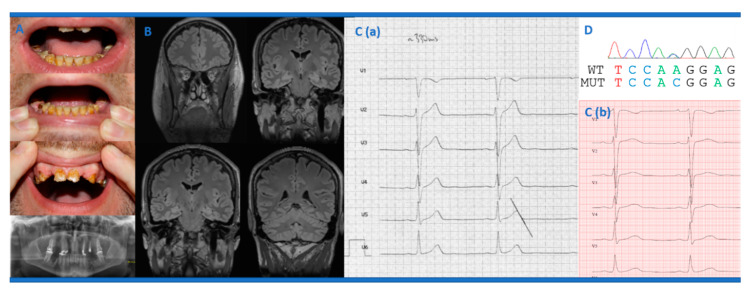
(**A**) The dental status at 29 years of age and the corresponding dental radiography showed a reduced density of several coronas and mild hypoplasia of the maxilla and mandible. (**B**) The cerebral magnetic resonance imaging of the patient revealed slight hippocampal damage on the left in the area of the corpus and the cauda, together with otherwise inconspicuous cerebral anatomic findings. (**C**) The baseline electrocardiography displayed a short QTc interval (heart rate 57/min, QT 330 ms, QTc: 324 ms; reference: > 350 ms; 3a) and normalization during treatment with quinidine (3 × 200 mg/d; QTc: 425 ms; reference: < 350 ms; 3b). (**D**) The DNA sequence electropherogram revealed a heterozygous state at position 2399 (c.2399A > C) in the *CACNA1C* gene predicted to result in p.Lys800Thr.

At the age of 15 years, the patient developed his first depressive episode and, at the age of 17 years, his first hypomanic episode. Until publication of this case report, the patient suffered from recurrent depressive episodes, but hypomanic phases did not occur anymore after the age of 21 years. His first psychiatric presentation took place at the age of 18 years following mutistic states (i.e., he was unable to speak); there were no indications of schizophrenia or causation by illegal drugs. Video telemetry revealed no evidence of epileptic seizures. At the age of 18 years, monomorphic ventricular extrasystoles (VES) were noticed for the first time during a physical examination for military service suitability. At the age of 29 years, the teeth suddenly discolored and then receded (Figure 1A). During this time, the patient suffered from significant polydipsia (requiring around 10 L of drinks/day) and was treated with lithium, methylphenidate, pregabalin, and lorazepam. Polydipsia was mainly caused by lithium treatment with increased serum levels at that time (1.6−1.8 mmol/L; reference 0.4–1.2 mmol/L). Calcium serum levels were within the normal range, and, thereby, phosphate and parathyroid levels were not determined. In the further course, 13 teeth had to be extracted and were supplemented by dental prostheses; other teeth were crowned. At the age of 29 years, he also developed a paranoid hallucinatory episode with hearing voices and ideas of persecution under the treatment with methylphenidate, nortriptyline, quetiapine, pregabalin, and lorazepam. Recurrently, the patient received benzodiazepines, but, after this was stopped (by antagonization with flumazenil), a generalized tonic-clonic seizure occurred at the age of 29 years.

Electroencephalography at age 29, 33, and 34 years was inconspicuous. Analysis of the cerebrospinal fluid (CSF) revealed a borderline blood-brain barrier dysfunction (with CSF protein levels of 579 mg/L; reference: < 450 mg/L). The immunological screening showed an antinuclear antibody (ANA) titer of 1:100 (reference: < 1:50) and an IgA deficiency (0.26 g/L; reference: 0.7−4 g/L).

A highly symptomatic occurrence of the monomorphic VES was noted again during the subsequent in-patient hospital treatment at the age of 29 years. Further cardiologic evaluation showed ~12,000 VES/day during Holter ECG, and subsequently, due to the severe overall clinical course, the patient underwent ablation therapy in the area of the middle cardiac vein or coronary sinus twice, which reduced VES burden (remaining: 3600 VES/day). There was no evidence of a structural heart disease as assessed by transthoracic echocardiography. The family history was positive with his father suffering from depression, alcohol-dependency, tachycardia (treated with ß-blockers), and unclear dental damage (the patient had no contact with the father; therefore, further information is missing). The age of the mother at birth of the patient was 41 years, and the age of the father was 35 years.

Over the years, due to the severe course of the psychiatric symptoms, multiple psychopharmacological treatment trials have been carried out using varying and sometimes high doses (sertraline, fluoxetine, escitalopram, paroxetine, venlafaxine, vortioxetine, bupropion, nortriptyline, amitriptyline, clomipramine, lithium, methylphenidate, haloperidol, aripiprazole, perazine, promethazine, quetiapine, valproate, lamotrigine, carbamazepine, oxcarbazepine, pregabalin, levetiracetam, zonisamide, lacosamide, lorazepam, clonazepam, oxazepam, clobazam).

After complete discontinuation of psychotropic medication at the age of 31 years, a cardiac re-evaluation was performed, and a short QTc interval of 324 ms (Figure 1C; reference: > 350 ms) was noted for the first time. Overall, this resembled a (paradoxical) shortening of the QTc interval during a heart rate (HR) of 57 bpm; at a HR of 111 bpm, the QTc interval normalized (408 ms). There was no evidence of T-wave alternans. Due to suspected SQTS, quinidine treatment (3 × 200 mg/day) has been initialized to normalize repolarization, but also to reduce symptomatic VES burden and thereby indirectly to improve mental strength and personal stability. Of note, during treatment QTc intervals completely recovered (at 57/min: QRS 114 ms, QTc around 425 ms; T-wave: broad-based, biphasic; no early repolarization signs or Brugada sign; Figure 1C), and the burden of symptomatic VES was nearly absent (now: 16 VES/day).

### 2.2. Genetic Analyses

Genetic testing of genes related to SQTS (*KCNQ1*, *KCNH2*, *KCNJ2*, *CACNA1C*) revealed a heterozygous nucleotide variant in *CACNA1C* (NM_000719.6: c.2399A > C; p.Lys800Thr), which was very rare in control databases (MAF gnomAD: 0.00043%) and predicted to affect protein function by 16 of the 23 pathogenicity prediction algorithms (VarCards). The mutated lysine is located in the cytoplasmic loop between repeat II and repeat III of the Cav1.2 calcium channel and is highly conserved. To exclude pathogenic copy number variations (CNV) as a possible additional cause of ASD and the psychiatric symptoms, microarray-analysis was performed (CytoSureTM Constitutional v3 Array 180k, Oxford Gene Technology) in the patient. Molecular karyotyping did not show any pathogenic or relevant CNVs.

### 2.3. Electrophysiological Analyses

**Figure 2:**
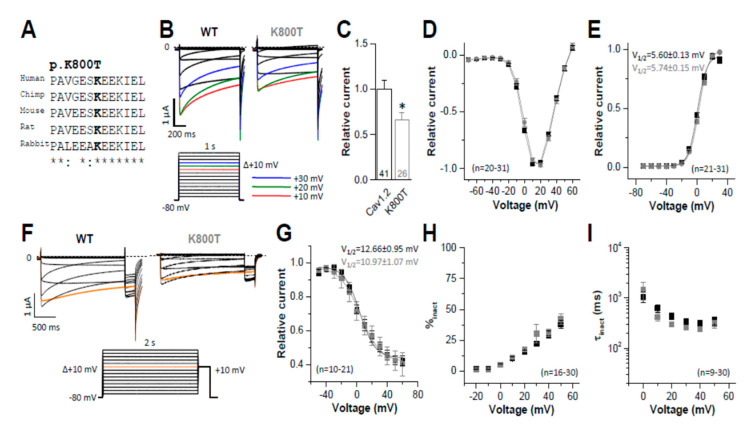
Own experimental analyses in the presented patient illustrated an isolated reduction in the current amplitude of L-type calcium channels without a change in kinetic properties. Two-electrode voltage-clamp measurements were performed in *Xenopus* oocytes, as previously described [2]. Ba^2+^ was used as a charge carrier, and for all experiments, Cav1.2 was co-expressed with the α2δ and β2b subunits [2]. In detail, the figure shows the following: (**A**) High evolutionary conservation of the K800 residue between different orthologues. (**B**) Representative current traces of wild-type (WT) Cav1.2 and mutant Cav1.2^K800T^ recorded with the indicated voltage protocol, in order to analyze the current amplitudes and the voltage-dependence of activation properties. (**C**) Reduced peak current amplitudes, analyzed at + 20 mV. (**D**) Normalized bell-shaped current voltage-relationship and (**E**) conductance-voltage (GV) relationship. The voltage of half-maximal activation (V1/2 act.) is indicated in the upper corner. (**F**) Representative current traces of wild-type Cav1.2 and Cav1.2^K800T^ recorded with the indicated voltage protocol, in order to analyze the inactivation properties. (**G**) Analyses of the voltage of half-maximal inactivation (V1/2 inact.). The V1/2 inact. values are provided in the upper corner. (**H**) Percentage of voltage-dependent inactivation of wild-type and Cav1.2^K800T^ analyzed at different voltage potentials. (**I**) Analyses of the time constant of voltage-dependent inactivation (τ inact.) of wild-type and Cav1.2^K800T^ analyzed at different voltage potentials. Numbers of experiments are indicted in the panels. Data are presented as mean ± s.e.m. * indicates *p* < 0.05.

Heterologous expression studies of the mutant Cav1.2 calcium channel in *Xenopus* oocytes showed an isolated reduction in the current amplitude of the L-type calcium channels (Figure 2B,C) without a change in kinetic properties (Figure 2D−I) when compared with native (wild-type) channels. Here, the normalized bell-shaped current-voltage relationship and the voltage-dependence of activation were unaltered (Figure 2D−E). In addition, the voltage-dependence of inactivation (Figure 2F−G), the extent of inactivation (Figure 2H), and the kinetics of inactivation (Figure 2I) were similar as in wild-type channels. Thus, the genetic variant is responsible for an isolated reduction of the macroscopic current amplitudes of approximately 40% (Figure 2C). The lack of changes in the channel kinetics and maintained voltage-dependence of channel gating behavior suggest that this reduced current amplitude is most likely caused by an intracellular trafficking defect of the mutant Cav1.2 calcium channel.

With respect to these functional data, the p.Lys800Thr variant was classified as likely pathogenic (class 4 variant) according to the ACMG guidelines [11].

## 3. Discussion

The authors present a patient with a previously unpublished loss-of-function mutation of the *CACNA1C* gene with a novel, syndromal phenotype characterized by ASD, affective and psychotic exacerbations, dental enamel defect, and a short QT interval in the surface ECG. 

The mutated *CACNA1C* gene (Chr. 12p13.3; 2,138 amino acids) encodes the α-subunit of the L-type calcium channel CaV1.2. These calcium channels are highly expressed in the brain and heart [3]. Genetic changes in *CACNA1C* are associated with Timothy syndrome or early repolarization disturbances/Brugada-like electrocardiography. However, patients with Timothy syndrome typically have gain-of-function mutations in the *CACNA1C* gene (e.g., p.Gly406Arg [2]), leading to maintained depolarizing L-type calcium current with long QT syndrome in the surface ECG and early sudden cardiac death (at an average age of 2.5 years; 3). Additionally, other cardiac features (AV conduction block with bradycardia, tachyarrhythmia, or congenital heart defects), neuropsychiatric involvement (developmental delays, seizures, ASD), hand/foot and facial findings (e.g., cutaneous syndactyly, low-set ears), hypoglycemia, and infections can be found in patients with Timothy syndrome [2]. Poor dental enamel was also reported earlier [3,12].

The presented case also suffered from high-functioning ASD, but he additionally presented with depressive and hypomanic episodes, as well as one paranoid hallucinatory episode. Such complex psychiatric syndromes are often found in patients with secondary, organic forms [1]. The genetic variant that has been classified finally as a likely pathogenic variant could explain the poor response to different psychotropic drugs in the patient’s history. However, pharmacogenetic studies, e.g., on drug metabolism, have not been carried out. There were no signs of dysmorphia on the hands/feet or face in the presented patient. In addition, he suffered from poor dental enamel with severe caries, initially misinterpreted as “meth mouth” because of high-dose methylphenidate medication. Surprisingly, a short QT interval was first noted when the patient was off psychotropic drugs that typically prolong the QT interval and, in this particular case, led to a normalization. He also had a high burden of VES. Only just about 250 cases with short QT syndrome are currently known, however, these have either isolated cardiac phenotypes or acquired, concomitant conditions (e.g., electrolyte disturbances or carnitine deficiency), and to date only about 30 genetic variants in eight potential disease genes were identified (summarized in [13]). Most of these cases are due to gain-of-function mutations in the potassium channel genes (*KCNH2, KCNQ1, and KCNJ2*), whereas loss-of-function mutations in *CACNA1C* leading to SQTS are extremely rare, and, in these cases, the shortened QT interval is accompanied with a Brugada ECG phenotype (not seen in the present case). Due to controversial or absent functional data and contradictory in silico predictions, some of variants were re-classified as variants of uncertain significance following ACMG criteria (class 3 variant) in a recent publication [14]. Very recently, a *CACNA1C* loss-of-function variant has been linked with SQTS in combination with early repolarization patterns in the surface ECG [15].

The heterozygous c.2399 A > C variant in the presented patient is yet unreported, leading to a non-synonymous amino acid exchange in the α-subunit of the L-type calcium channel CaV1.2 (p.(Lys800Thr)). A causality of the mutation is possible because the gene mutation is located in a conserved gene region and is very rare in control databases. Most pathogenicity prediction programs that evaluate amino acid exchange in silico suggest a biochemical and thus pathogenic effect (VarCards: 16/23) as it has been shown by the in vitro data upon heterologous expression experiments. The variant finally could be classified as a class 4 variant (“likely pathogenic”) according to the ACMG/AMP guideline [11]. Our functional studies revealed an isolated reduction of the current amplitudes (Figure 2C) without changes in the gating behavior of the channel. Therefore, a weak trafficking defect is the most likely mechanism of action for this atypical Cav1.2 channelopathy with brain, cardiac and dental enamel involvement.

## 4. Conclusions

In summary, this is a paradigmatic case of a secondary genetic variant of a complex, partially atypical mental disorder with heart and dental involvement. Many such secondary genetic variants probably occur, each of which is very rare, but, taken together, these rare variants might be causing a relevant subgroup of mental disorders. In the presented patient, a pathological variant can be assumed, which led to an atypical form of SQTS with a clinical spectrum that partially overlaps with that of Timothy syndrome. Associations between CaV1.2 channel (*CACNA1C*) polymorphisms and different psychiatric disorders, including autism, depression, bipolar disorder, and schizophrenia, were recently reported [16,17,18,19]. Further research will need to show whether similar cases exist and elucidate the therapeutic consequences that can be inferred from mutant Cav1.2 channel.
